# Adverse events following immunisation with a meningococcal serogroup B vaccine: report from post-marketing surveillance, Germany, 2013 to 2016

**DOI:** 10.2807/1560-7917.ES.2018.23.17.17-00468

**Published:** 2018-04-26

**Authors:** Dirk Mentzer, Doris Oberle, Brigitte Keller-Stanislawski

**Affiliations:** 1 Department Safety of Medicinal Products and Medical Devices, Paul-Ehrlich-Institut, Federal Institute for Vaccines and Biomedicines, Langen, Germany; 2 DM and DO contributed equally to this article

**Keywords:** 4CMenB, adverse events following immunisation, Bexsero, invasive meningococcal disease, Neisseria meningitidis serogroup B, safety profile, spontaneous reporting, vaccine safety

## Abstract

In January 2013, a novel vaccine against *Neisseria meningitidis* serogroup B, the multicomponent meningococcal serogroup B vaccine (4CMenB), was approved by the European Medicines Agency. We aimed to evaluate the safety profile of this vaccine. **Methods:** All adverse events following immunisation (AEFI) reported from Germany since the vaccine’s launch in Germany in November 2013 through December 2016 were reviewed and analysed. **Results:** Through December 2016, a total of 664 individual case safety reports (ICSR) notifying 1,960 AEFI were received. A majority of vaccinees for whom AEFI were reported were children 2 to 11 years of age (n = 280; 42.2%) followed by infants and toddlers aged 28 days to 23 months (n = 170; 25.6%). General disorders and administration site conditions was the System Organ Class (SOC) with the majority of AEFI (n = 977; 49.8%), followed by nervous system disorders (n = 249; 12.7%), and skin and subcutaneous tissue disorders (n = 191; 9.7%). Screening of patient records for immune-mediated and neurological diseases did not raise any safety signal in terms of an increased proportional reporting ratio (PRR). **Conclusions:** The safety profile described in the Summary of Product Characteristics, in general, is confirmed by data from spontaneous reporting. No safety concerns were identified.

## Introduction

Infection with *Neisseria meningitidis*, an aerobic encapsulated Gram-negative diplococcus, may be life-threatening or result in major long-term sequelae.

In 2015, within the scope of a surveillance programme coordinated by the European Centre of Disease Prevention and Control (ECDC), the notification rate of invasive meningococcal disease was 0.6 cases per 100,000 population (lower and upper bound: 0.1–2.0) in the European Union/European Economic Area (EU/EEA) and 0.4 cases per 100,000 population in Germany [1]. Particularly high age-specific rates in the EU/EEA were found in infants under one year of age at 10.0 cases per 100,000 population and in children 1 to 4 years of age at 2.8 cases per 100,000 population [1]. The majority of cases with a known serogroup belonged to serogroup B (61%) [1].

Until 2012, no broadly effective serogroup B meningococcal vaccines were available as the capsular polysaccharide of meningococcal serogroup B is poorly immunogenic in humans [2,3]. This is why research has focused on proteins in the outer membrane of meningococci as potential antigens for candidate vaccines [2,3].

The multicomponent meningococcal serogroup B vaccine (4CMenB), Bexsero (GSK Vaccines S.r.l., Siena, Italy), contains four antigenic components: factor H binding protein, Neisseria adhesin A, Neisseria heparin-binding antigen and outer membrane vesicles from a New Zealand epidemic strain that produces Porin A, the immunodominant antigen that is present in the outer membrane vesicle component [4].

We aimed to evaluate and complement the safety profile of 4CMenB as described in the Summary of Product Characteristics. A special focus was placed on immune-mediated and severe neurological outcomes.

## Methods

Adverse events following immunisation (AEFI) with 4CMenB reported in Germany since the vaccine’s launch on the German market on 27 November 2013 through 31 December 2016 were reviewed.

### Spontaneous reporting

Germany has a mandatory reporting system for AEFI that is used for vaccine safety surveillance. According to §6(3) of the Protection against Infection Act (Infektionsschutzgesetz, IfSG) [5], it is mandatory for healthcare professionals to report AEFI to the local health authorities which themselves are obliged to forward the notification to the national competent authority. Marketing authorisation holders have to report suspected serious adverse reactions directly to the national competent authority according to §63c of the German Medicinal Products Act (Arzneimittelgesetz, AMG) [6]. In addition, vaccinees or their relatives may notify AEFI, so-called consumer reports, via an online database. The seriousness of individual case safety reports (ICSR) was determined according the International Council for Harmonisation (ICH) Topic E 2 A guideline [7]. We reviewed all ICSR received from 2013 to 2016, including consumer reports.

After the launch of 4CMenB in Germany in November 2013, the national competent authority agreed with the marketing authorisation holder on a monthly expedited reporting of non-serious AEFI in addition to the expedited reporting of serious AEFI.

All AEFI included in ICSR were coded by trained data entry staff according to the Medical Dictionary for Regulatory Activities (MedDRA) [8] in Lowest Level Terms (LLTs), the coding level that provides maximum specificity. In MedDRA terminology, selection of a LLT leads to automatic assignment of grouping terms higher in the hierarchy: Preferred Terms (PTs), High Level Terms (HLTs), High Level Group Terms (HLGTs) and System Organ Classes (SOCs).

### Definition of age groups

Age groups concerning individuals 17 years of age and under were defined according to ICH guideline, Clinical Investigation of Medicinal Products in the Paediatric Population E11 [9]: newborns (0 to 27 days of age), infants and toddlers (28 days to 23 months of age), children (2 to 11 years of age) and adolescents (12 to 17 years of age). Adults were stratified into two groups: individuals 18 to 59 years of age and those 60 years of age and over.

### Denominator

Vaccines have to undergo batch release testing before they can be marketed. Thus, the number of doses released by the national competent authority for the German market from the vaccine’s launch through December 2016 was used as a surrogate for the number of doses administered.

### Descriptive analysis, reporting rates and proportional reporting ratios

For qualitative variables, absolute and relative frequencies were calculated. For quantitative variables, medians, minimums and maximums were computed. In addition, stratification by age group was performed. Reporting rates, the number of specific AEFI divided by the number of doses released, were calculated for PTs with a count of at least three.

For the 10 most frequently coded PTs, disproportionality analyses were performed by calculating proportional reporting ratios (PRR) [10] and 95% confidence intervals (95% CI). Evans et al. [10] defined three minimum criteria for a safety signal: three or more cases, a PRR of at least two and a chi-squared of at least four. For comparison with other products used in routine immunisation with respect to these PTs, PRRs and 95% CI were calculated for meningococcal vaccines (C plus combinations other than type B) and pneumococcal conjugate vaccines.

### Screening of patient records for immune-mediated and neurological diseases

In order to screen for safety signals with respect to immune-mediated and neurological diseases, we used a list of 52 event outcomes published by Arnheim-Dahlström et al. [11] and calculated PRRs for them.

### Identifying adverse events of specific interest

Furthermore, PRRs and 95% CI were also calculated for selected adverse events of specific interest: febrile convulsion/seizure, anaphylactic reaction/shock, hypotonic-hyporesponsive episode and apparent life-threatening event.

### Case definitions and causality assessment

For immune-mediated and neurological outcomes as well as for adverse events of specific interest, case definitions published by the Brighton Collaboration [12], if available, were used for case validation according to diagnostic certainty.

Causality of ICSR and AEFI was assessed according to the revised World Health Organization (WHO) classification [13]. If adequate information for causality conclusion was available, the assessment of an ICSR/AEFI according to the algorithm described in Step 3 and Figure 3 of the WHO classification document was ‘consistent causal association to immunisation’ or ‘inconsistent causal association to immunisation’. The association was assessed as ‘indeterminate’ when adequate information was available but it was impossible to assign an ICSR/AEFI to either of the aforementioned categories. If adequate information was not available, the assessment was ‘unclassifiable’.

### Statistical analysis

The statistical analysis was performed using the SAS version 9.4 (SAS Institute, Cary, NC, United States).

## Results

Through December 2016, a total of 664 ICSR were received, 137 of which (20.6%) were classified as serious. In most of the ICSR (n = 626; 94.3%), 4CMenB was administered without concomitant vaccines. The majority of notifications (n = 600; 90.4%) originated from the marketing authorisation holders, while 49 (7.4%) originated from healthcare professionals and 15 (2.3%) from consumers.

Two-hundred-and-ninety vaccinees with reported AEFI were males (43.7%), 321 were females (48.3%) and sex was unknown for 53 (8.0%). The median age was 5.0 years (range: 44 days–69 years). The majority of ICSR concerned children 2 to 11 years of age (n = 280; 42.2%) as well as infants and toddlers aged 28 days to 23 months (n = 170; 25.6%) (Table 1).

**Table 1:** Reporting year and demographic characteristics of multicomponent meningococcal serogroup B vaccine (4CMenB) recipients addressed in individual case safety reports (ICSR), Germany, 2013–2016 (n = 664)

Reporting year and sex	Age group												Total	
	28 days–23 months^a^		2–11 years		12–17 years		18–59 years		≥ 60 years		NA			
	n	%	n	%	n	%	n	%	n	%	n	%	n	%
**Reporting year**														
2013	1	0.6	0	0.0	0	0.0	0	0.0	0	0.0	1	1.7	2	0.3
2014	47	27.6	97	34.6	22	37.3	41	44.1	1	50.0	31	51.7	239	36.0
2015	48	28.2	97	34.6	14	23.7	19	20.4	0	0.0	16	26.7	194	29.2
2016	74	43.5	86	30.7	23	39.0	33	35.5	1	50.0	12	20.0	229	34.5
**Sex**														
Male	76	44.7	140	50.0	22	37.3	29	31.2	0	0.0	23	38.3	290	43.7
Female	86	50.6	118	42.1	35	59.3	61	65.6	2	100	19	31.7	321	48.3
NA	8	4.7	22	7.9	2	3.4	3	3.2	0	0.0	18	30.0	53	8.0
**Total**	**170**	**100**	**280**	**100**	**59**	**100**	**93**	**100**	**2**	**100**	**60**	**100**	**664**	**100**

NA: not available.

^a^ There was one medication error where vaccine was administered to an infant that was too young, being 44 days of age at time of vaccination.

### Outcomes and assessment of causality

In 358 ICSR (53.9%), the outcome at the time of reporting was ‘recovered’, in 25 ICSR (3.8%) ‘improved’, in 65 ICSR (9.8%) ‘not recovered’, and in 214 ICSR (32.2%) ‘unknown’. In one case (0.2%) the vaccinee suffered permanent damage and in another case (0.2%) the vaccinee died (Table 2). Of the ICSR, 452 (68.1%) were assessed as ‘consistent’ and 50 (7.5%) as ’inconsistent’ to a causal association to immunisation. For 17 ICSR (2.6%), causality was considered ‘indeterminate’ and in 145 ICSR (21.8%), ‘unclassifiable’ (Table 2).

**Table 2:** Outcome and causality assessment of individual case safety reports (ICSR) following immunisation with multicomponent meningococcal serogroup B vaccine (4CMenB) by age group, Germany, 2013–2016 (n = 664)

Outcome and causality assessment	Age group												Total	
	28 days–23 months		2–11 years		12–17 years		18–59 years		≥ 60 years		NA			
	n	%	n	%	n	%	n	%	n	%	n	%	n	%
**Outcome^a^**														
Recovered	94	55.3	151	53.9	34	57.6	53	57.0	1	50.0	25	41.7	358	53.9
Improved	6	3.5	13	4.6	1	1.7	5	5.4	0	0.0	0	0.0	25	3.8
Not recovered	24	14.1	25	8.9	2	3.4	10	10.8	1	50.0	3	5.0	65	9.8
Sequelae	1	0.6	0	0.0	0	0.0	0	0.0	0	0.0	0	0.0	1	0.2
Death	0	0.0	1	0.4	0	0.0	0	0.0	0	0.0	0	0.0	1	0.2
Unknown	45	26.5	90	32.1	22	37.3	25	26.9	0	0.0	32	53.3	214	32.2
**Causality assessment^b^**														
Consistent	123	72.4	189	67.5	41	69.5	59	63.4	2	100	38	63.3	452	68.1
Indeterminate	5	2.9	9	3.2	0	0.0	2	2.2	0	0.0	1	1.7	17	2.6
Inconsistent	11	6.5	21	7.5	2	3.4	13	14.0	0	0.0	3	5.0	50	7.5
Unclassifiable	31	18.2	61	21.8	16	27.1	19	20.4	0	0.0	18	30.0	145	21.8
**Total**	**170**	**100**	**280**	**100**	**59**	**100**	**93**	**100**	**2**	**100**	**60**	**100**	**664**	**100**

NA: not available.

^a^ At the date of reporting.

^b^ Causality of an ICSR was assessed according to the revised World Health Organization (WHO) classification [13].

#### Cases that resulted in sequelae or death

There were two cases that resulted in sequelae or death. The case that resulted in sequelae was a male infant 5 months of age who received the first dose intramuscularly in the left lateral thigh. On the same day, the patient developed subcutaneous injection site abscess that was livid, persistent and fluctuating. The abscess was surgically treated, leaving behind a scar with lipolysis ca 2 x 2.5 cm. This case report was assessed as ‘consistent’ to a causal association to the administration of the vaccine.  The one ICSR with a fatal outcome was a male child 28 months of age whose family history included long QT syndrome. The patient was born after induction of labour in the 39th week of pregnancy because of oligohydramnion and placental insufficiency. Medical history included a middle ear inflammation 4 months before the child’s death, dry skin and cough hypersensitivity syndrome. Previous receipt of 4CMenB administered at the age of 25 months was well tolerated. Seventeen days after intramuscular administration of the second dose, and with no concomitant medication reported, the child was affectionate and complained about throat pain in the early morning. In the late morning, the child was found lifeless lying in bed with his head on the pillow. Cardiopulmonary resuscitation was performed for 1 hour without success. Autopsy could not identify any cause of death. On the basis of the WHO algorithm for causality assessment of AEFI, this case report was classified as ‘inconsistent’ to a causal association to immunisation.

### Time to symptoms onset

The time to symptoms onset ranged from the day of vaccination to 81 days after vaccination with the median time to symptoms onset being 0 days, i.e. less than 24 hours (data not shown). Regarding time to symptoms onset, differences between the age groups were negligible.

### Adverse events

In Table 3, the 1,960 AEFI reported in the 664 ICSR were stratified by SOC and age group. General disorders and administrations site conditions was the SOC with the majority of AEFI (n = 977; 49.8%) followed by the SOCs nervous system disorders (n = 249; 12.7%), and skin and subcutaneous tissue disorders (n = 191; 9.7%). The distribution of AEFI on the SOCs was similar in all age groups. However, there was some variability regarding the HLGTs within a SOC. Within the SOC ‘General disorders and administration site conditions’, there was a higher percentage of body temperature conditions in infants and toddlers (77/195; 39.5%) and children (111/442; 25.1%) than in adolescents (10/85; 11.8%) and adults 18–59 years of age (10/173; 5.8%). Similarly, regarding the SOC ‘Nervous system disorders’, there was a higher percentage of headaches in adults 18–59 years of age (19/47) and adolescents (12/24) than in children (24/86; 27.9%) and infants and toddlers (0/68; 0.0%). However, there was a higher percentage of seizures in infants and toddlers (16/68; 23.5%) and children (10/86; 11.6%) than in adolescents (0/24) and adults 18–59 years of age (1/47).

**Table 3:** Adverse events following immunisation (AEFI) with multicomponent meningococcal serogroup B vaccine (4CMenB) by System Organ Class (SOC) and age group, Germany, 2013–2016 (n = 1,960)

SOC	Age group												Total	
	28 days–23 months		2–11 years		12–17 years		18–59 years		≥ 60 years		NA			
	n	%	n	%	n	%	n	%	n	%	n	%	n	%
General disorders and administration site conditions	195	41.6	442	53.9	85	48.0	173	53.2	2	66.7	80	48.2	977	49.8
Nervous system disorders	68	14.5	86	10.5	24	13.6	47	14.5	1	33.3	23	13.9	249	12.7
Skin and subcutaneous tissue disorders	47	10.0	90	11.0	13	7.3	29	8.9	0	0.0	12	7.2	191	9.7
Musculoskeletal and connective tissue disorders	19	4.1	58	7.1	22	12.4	36	11.1	0	0.0	15	9.0	150	7.7
Gastrointestinal disorders	17	3.6	37	4.5	12	6.8	10	3.1	0	0.0	7	4.2	83	4.2
Psychiatric disorders	46	9.8	19	2.3	3	1.7	3	0.9	0	0.0	4	2.4	75	3.8
Investigations	10	2.1	19	2.3	3	1.7	5	1.5	0	0.0	2	1.2	39	2.0
Infections and infestations	14	3.0	14	1.7	0	0.0	4	1.2	0	0.0	4	2.4	36	1.8
Vascular disorders	12	2.6	14	1.7	5	2.8	2	0.6	0	0.0	0	0.0	33	1.7
Injury, poisoning and procedural complications	5	1.1	11	1.3	2	1.1	4	1.2	0	0.0	5	3.0	27	1.4
Respiratory, thoracic and mediastinal disorders	11	2.3	6	0.7	2	1.1	0	0.0	0	0.0	6	3.6	25	1.3
Cardiac disorders	8	1.7	4	0.5	3	1.7	3	0.9	0	0.0	0	0.0	18	0.9
Blood and lymphatic system disorders	1	0.2	4	0.5	0	0.0	5	1.5	0	0.0	4	2.4	14	0.7
Metabolism and nutrition disorders	8	1.7	5	0.6	0	0.0	1	0.3	0	0.0	0	0.0	14	0.7
Eye disorders	3	0.6	6	0.7	1	0.6	1	0.3	0	0.0	0	0.0	11	0.6
Product issues	0	0.0	0	0.0	2	1.1	2	0.6	0	0.0	1	0.6	5	0.3
Ear and labyrinth disorders	2	0.4	1	0.1	0	0.0	0	0.0	0	0.0	1	0.6	4	0.2
Renal and urinary disorders	1	0.2	1	0.1	0	0.0	0	0.0	0	0.0	1	0.6	3	0.2
Immune system disorders	0	0.0	2	0.2	0	0.0	0	0.0	0	0.0	0	0.0	2	0.1
Social circumstances	0	0.0	1	0.1	0	0.0	0	0.0	0	0.0	1	0.6	2	0.1
Neoplasms benign, malignant and unspecified (including cysts and polyps)	1	0.2	0	0.0	0	0.0	0	0.0	0	0.0	0	0.0	1	0.1
Pregnancy, puerperium and perinatal conditions	1	0.2	0	0.0	0	0.0	0	0.0	0	0.0	0	0.0	1	0.1
**Total**	**469**	**100**	**820**	**100**	**177**	**100**	**325**	**100**	**3**	**100**	**166**	**100**	**1,960**	**100**

NA: not available.

Stratification by SOC and sex did not reveal any relevant differences between males and females (data not shown).

The overall reporting rate was 244.8 AEFI per 100,000 doses released.

In Table 4, PTs that were coded at least three times are shown grouped under HLTs, HLGTs and SOCs, representing 86.0% of all AEFI (n = 1,685).

**Table 4:** Preferred Terms (PT) for adverse events following immunisation (AEFI) with multicomponent meningococcal serogroup B vaccine (4CMenB) that were coded at least three times, grouped under High Level Terms (HLT), High Level Group Terms (HLGT) and System Organ Class (SOC), Germany, 2013–2016 (n =1,685)

SOC	HLGT	HLT	PT	n	Relative frequency (%)^a^	Reporting rate (reported number of AEFI per 100,000 doses released)
Blood and lymphatic system disorders	Spleen, lymphatic and reticuloendothelial system disorders	Lymphatic system disorders NEC	Lymphadenopathy	11	0.6	1.4
Cardiac disorders	Cardiac disorder signs and symptoms	Cardiac disorders NEC	Cardiovascular disorder	6	0.3	0.7
		Cardiac signs and symptoms NEC	Cyanosis	9	0.5	1.1
Eye disorders	Vision disorders	Visual disorders NEC	Visual impairment	3	0.2	0.4
Gastrointestinal disorders	Gastrointestinal motility and defaecation conditions	Diarrhoea (excluding infective)	Diarrhoea	14	0.7	1.7
	Gastrointestinal signs and symptoms	Gastrointestinal and abdominal pains (excluding oral and throat)	Abdominal pain	6	0.3	0.7
		Nausea and vomiting symptoms	Nausea	23	1.2	2.9
			**Vomiting**	**30**	**1.5**	**3.7**
General disorders and administration site conditions	Administration site reactions	Injection site reactions	Extensive swelling of injected limb	21	1.1	2.6
			Injected limb mobility decreased	27	1.4	3.4
			Injection site discolouration	9	0.5	1.1
			Injection site discomfort	6	0.3	0.7
			Injection site erythema	11	0.6	1.4
			Injection site granuloma	17	0.9	2.1
			Injection site haematoma	4	0.2	0.5
			**Injection site induration**	**55**	**2.8**	**6.9**
			Injection site mass	3	0.2	0.4
			Injection site movement impairment	5	0.3	0.6
			**Injection site pain**	**131**	**6.7**	**16.4**
			Injection site reaction	15	0.8	1.9
			**Injection site swelling**	**109**	**5.6**	**13.6**
			Injection site warmth	27	1.4	3.4
	Body temperature conditions	Febrile disorders	Hyperpyrexia	3	0.2	0.4
			**Pyrexia**	**219**	**11.2**	**27.4**
	General system disorders NEC	Asthenic conditions	Asthenia	17	0.9	2.1
			**Fatigue**	**29**	**1.5**	**3.6**
			Malaise	20	1.0	2.5
		Feelings and sensations NEC	Chills	24	1.2	3.0
			Feeling abnormal	4	0.2	0.5
		Gait disturbances	Abasia	8	0.4	1.0
			Gait disturbance	10	0.5	1.2
		General signs and symptoms NEC	Crying	22	1.1	2.7
			General physical health deterioration	12	0.6	1.5
			Induration	4	0.2	0.5
			Influenza-like illness	11	0.6	1.4
			Local reaction	18	0.9	2.2
			Local swelling	5	0.3	0.6
			Peripheral swelling	9	0.5	1.1
			Swelling	22	1.1	2.7
		Inflammations	Granuloma	10	0.5	1.2
			Inflammation	5	0.3	0.6
		Pain and discomfort NEC	**Pain**	**43**	**2.2**	**5.4**
			Tenderness	4	0.2	0.5
Infections and infestations	Infections (pathogen unspecified)	Infections NEC	Infection	4	0.2	0.5
		Lower respiratory tract and lung infections	Pneumonia	4	0.2	0.5
		Upper respiratory tract infections	Nasopharyngitis	4	0.2	0.5
		Vascular infections	Lymphangitis	3	0.2	0.4
Injury, poisoning and procedural complications	Injuries NEC	Non-site specific injuries NEC	Fall	3	0.2	0.4
	Medication errors	Maladministrations	Expired product administered	5	0.3	0.6
			Inappropriate schedule of drug administration	7	0.4	0.9
Investigations	Physical examination and organ system status topics	Physical examination procedures and organ system status	Body temperature increased	14	0.7	1.7
	Protein and chemistry analyses NEC	Protein analyses NEC	C-reactive protein increased	6	0.3	0.7
Metabolism and nutrition disorders	Appetite and general nutritional disorders	Appetite disorders	Decreased appetite	5	0.3	0.6
			Diet refusal	3	0.2	0.4
	Electrolyte and fluid balance conditions	Fluid intake decreased	Fluid intake reduced	3	0.2	0.4
Musculoskeletal and connective tissue disorders	Joint disorders	Arthropathies NEC	Arthritis	5	0.3	0.6
		Joint related signs and symptoms	Joint swelling	3	0.2	0.4
	Muscle disorders	Muscle infections and inflammations	Myositis	4	0.2	0.5
		Muscle pains	Myalgia	17	0.9	2.1
		Muscle related signs and symptoms NEC	Muscle spasms	4	0.2	0.5
			Muscle twitching	5	0.3	0.6
		Muscle weakness conditions	Muscular weakness	8	0.4	1.0
	Musculoskeletal and connective tissue disorders NEC	Musculoskeletal and connective tissue pain and discomfort	Limb discomfort	4	0.2	0.5
			Neck pain	3	0.2	0.4
			**Pain in extremity**	**64**	**3.3**	**8.0**
		Musculoskeletal and connective tissue signs and symptoms NEC	Mobility decreased	6	0.3	0.7
			Musculoskeletal stiffness	9	0.5	1.1
Nervous system disorders	Headaches	Headaches NEC	**Headache**	**57**	**2.9**	**7.1**
	Movement disorders (including Parkinsonism)	Dyskinesias and movement disorders NEC	Movement disorder	7	0.4	0.9
		Paralysis and paresis (excluding cranial nerve)	Monoplegia	3	0.2	0.4
		Tremor (excluding congenital)	Tremor	4	0.2	0.5
	Neurological disorders NEC	Coordination and balance disturbances	Nystagmus	3	0.2	0.4
		Disturbances in consciousness NEC	Loss of consciousness	9	0.5	1.1
			Somnolence	7	0.4	0.9
			Syncope	8	0.4	1.0
		Neurological signs and symptoms NEC	Dizziness	17	0.9	2.1
			Eye movement disorder	4	0.2	0.5
			Meningism	5	0.3	0.6
			Myoclonus	4	0.2	0.5
			Unresponsive to stimuli	3	0.2	0.4
		Paraesthesias and dysaesthesias	Hyperaesthesia	9	0.5	1.1
			Hypoaesthesia	4	0.2	0.5
			Paraesthesia	6	0.3	0.7
	Neuromuscular disorders	Muscle tone abnormal	Hypotonia	13	0.7	1.6
		Neuromuscular disorders NEC	Hypotonic-hyporesponsive episode	3	0.2	0.4
	Seizures (including subtypes)	Seizures and seizure disorders NEC	Febrile convulsion	12	0.6	1.5
			Seizure	8	0.4	1.0
	Sleep disturbances (including subtypes)	Narcolepsy and hypersomnia	Hypersomnia	3	0.2	0.4
Product issues	Product quality, supply, distribution, manufacturing and quality system issues	Product quality issues NEC	Product quality issue	4	0.2	0.5
Psychiatric disorders	Anxiety disorders and symptoms	Anxiety symptoms	Anxiety	3	0.2	0.4
	Changes in physical activity	Increased physical activity levels	Restlessness	13	0.7	1.6
	Communication disorders and disturbances	Speech articulation and rhythm disturbances	Screaming	9	0.5	1.1
	Depressed mood disorders and disturbances	Mood alterations with depressive symptoms	Depressed mood	3	0.2	0.4
	Mood disorders and disturbances NEC	Emotional and mood disturbances NEC	Irritability	6	0.3	0.7
		Mood disorders NEC	Apathy	7	0.4	0.9
	Psychiatric and behavioural symptoms NEC	Abnormal behaviour NEC	Abnormal behaviour	3	0.2	0.4
	Sleep disorders and disturbances	Disturbances in initiating and maintaining sleep	Insomnia	3	0.2	0.4
		Sleep disorders NEC	Sleep disorder	6	0.3	0.7
Respiratory, thoracic and mediastinal disorders	Respiratory disorders NEC	Breathing abnormalities	Dyspnoea	3	0.2	0.4
		Coughing and associated symptoms	Cough	3	0.2	0.4
		Upper respiratory tract signs and symptoms	Rhinorrhoea	3	0.2	0.4
	Upper respiratory tract disorders (excluding infections)	Pharyngeal disorders (excluding infections and neoplasms)	Pharyngeal erythema	3	0.2	0.4
Skin and subcutaneous tissue disorders	Angioedema and urticaria	Urticarias	Urticaria	24	1.2	3.0
	Epidermal and dermal conditions	Bullous conditions	Blister	7	0.4	0.9
		Dermal and epidermal conditions NEC	Skin discolouration	4	0.2	0.5
		Erythemas	**Erythema**	**100**	**5.1**	**12.5**
		Pruritus NEC	Pruritus	6	0.3	0.7
		Rashes, eruptions and exanthems NEC	Rash	11	0.6	1.4
			Rash generalised	10	0.5	1.2
			Rash maculo-papular	3	0.2	0.4
	Skin vascular abnormalities	Purpura and related conditions	Henoch-Schoenlein purpura	3	0.2	0.4
Vascular disorders	Decreased and non-specific blood pressure disorders and shock	Circulatory collapse and shock	Circulatory collapse	3	0.2	0.4
			Peripheral circulatory failure	3	0.2	0.4
		Vascular hypotensive disorders	Hypotension	3	0.2	0.4
	Vascular disorders NEC	Peripheral vascular disorders NEC	Peripheral vascular disorder	4	0.2	0.5
		Site-specific vascular disorders NEC	Pallor	12	0.6	1.5
Total for all reactions				1,685	86.0	210.4

HLGT: High Level Group Terms; HLT: High Level Terms; NEC: not elsewhere classified; PT: Preferred Terms; SOC: System Organ Class.

^a^ Denominator is all reported AEFI (n = 1,960).

The ten most frequently coded Preferred Terms (PT) are in bold.

The 10 most frequently coded PTs, including PRRs, are presented in Table 5. Compared with meningococcal C ( plus combinations) or pneumococcal conjugate vaccines, higher PRRs were calculated for 4CMenB particularly with respect to local reactions (injection site induration, injection site swelling and pain responses) and pyrexia. This pattern was seen in all age groups (data not shown).

**Table 5:** Comparison of the ten most frequently coded Preferred Terms (PT) following immunisation with multicomponent meningococcal serogroup B vaccine (4CMenB) with meningococcal C (plus combinations) and pneumococcal conjugate vaccines, Germany, 2013–2016

Preferred Term	4CMenB	Meningococcal C (plus combinations) vaccines	Pneumococcal conjugate vaccines
Pyrexia	**n = 219; PRR 2.00 (1.76–2.27);** **χ^2^ = 111.90**	n = 186; PRR 1.36 (1.18–1.56); χ^2^ = 18.09	n = 265; PRR 1.24 (1.10–1.39); χ^2^ = 12.21
Injection site pain	**n = 131; PRR 3.88 (3.27–4.60);** **χ^2^ = 268.65**	n = 30; PRR 0.68 (0.48–0.98); χ^2^ = 4.46	n = 70; PRR 1.02 (0.81–1.30); χ^2^ = 0.04
Injection site swelling	**n = 109; PRR 3.50 (2.90–4.22);** **χ^2^ = 187.27**	n = 61; PRR 1.54 (1.20–1.98); χ^2^ = 11.30	**n = 124; PRR 2.04 (1.71–2.44);** **χ^2^ = 62.70**
Erythema	n = 100; PRR 1.60 (1.31–1.94); χ^2^ = 22.36	n = 131; PRR 1.69 (1.43–2.01); χ^2^ = 37.26	n = 231; PRR 1.94 (1.70–2.20); χ^2^ = 102.08
Pain in extremity	n = 64; PRR 1.63 (1.28–2.09); χ^2^ = 15.69	n = 27; PRR 0.55 (0.37–0.80); χ^2^ = 10.25	n = 36; PRR 0.46 (0.33–0.64); χ^2^ = 22.67
Headache	n = 57; PRR 1.07 (0.83–1.39); χ^2^ = 0.28	n = 79; PRR 1.20 (0.97–1.50); χ^2^ = 2.73	n = 17; PRR 0.16 (0.10–0.26); χ^2^ = 76.28
Injection site induration	**n = 55; PRR 7.89 (5.98–10.41);** **χ^2^ = 295.09**	n = 12; PRR 1.26 (0.71– 2.23); χ^2^ = 0.63	n = 28; PRR 1.92 (1.32–2.81); χ^2^ = 11.75
Pain	**n = 43; PRR 2.39 (1.77–3.24);** **χ^2^ = 33.95**	n = 13; PRR 0.57 (0.33–0.98); χ^2^ = 4.28	n = 43; PRR 1.22 (0.90–1.65); χ^2^ = 1.62
Vomiting	n = 30; PRR 1.07 (0.75–1.53); χ^2^ = 0.14	n = 40; PRR 1.16 (0.85–1.58); χ^2^ = 0.84	n = 55; PRR 1.01 (0.78– 1.32); χ^2^ = 0.01
Fatigue	n = 29; PRR 1.07 (0.75–1.54) ; χ^2^ = 0.14	n = 30; PRR 0.89 (0.62–1.28); χ^2^ = 0.38	n = 28; PRR 0.53 (0.36–0.76); χ^2^ = 11.93

PRR: proportional reporting ratio (95% confidence intervals); χ^2^: chi-squared.

Potential safety signals according to Evans et al. [10] in bold.

### Screening of patient records for safety signals for immune-mediated and neurological diseases

Notifications included six of 40 immune-mediated and three of 12 neurologic outcomes specified by Arnheim-Dahlström et al. [11]. For none of the outcomes, disproportionality measures were raised (data not shown).

ICSR reporting of suspected immune-mediated or neurological diseases concerned 12 individuals, seven males and five females, aged 5 months to 45 years at the time of AEFI. Three of these individuals had underlying conditions (Table 6).

**Table 6:** Immune-mediated and neurological^a^ diseases following immunisation with multicomponent meningococcal serogroup B vaccine (4CMenB), Germany, 2013–2016

Preferred term	Preferred term notification number	Sex	Age	Underlying conditions/concomitant diseases	Previous vaccination with 4CMenB	Time to symptom onset	BC case classification fulfilled	Outcome at the date of reporting	Causality assessment^b^	Comments
**Immune-mediated diseases** ^a^										
Henoch-Schoenlein purpura	1	M	5 years	None	No	8 days	NAP	Recovered	Inconsistent	^c^
	2	M	13 years	None	No	NA	NAP	Recovered	Unclassifiable	^d^
	3	F	4 years	Trichotillomania	No	3 days	NAP	Recovered	Unclassifiable	Infection, rhinitis and cough about 2 weeks before vaccination
Kawasaki’s disease	1	M	5 years	None	No	NA	NAP	Unknown	Inconsistent	^c^
Vasculitis unspecified	1	M	13 years	None	No	NA	NAP	Recovered	Unclassifiable	^d^
Juvenile idiopathic arthritis	1	F	5 years	None	Yes	6 days	NAP	Not recovered	Indeterminate	No comments
Myositis	1	F	5 months	None	Yes	1 day	NAP	Not recovered	Consistent	Abscess excluded
	2	F	4 years	NA	Yes	14 days	NAP	Recovered	Consistent	No comments
	3	M	41 years	NA	No	NA	NAP	Unknown	Indeterminate	^e^
	4	M	41 years	NA	Yes	NA	NAP	Unknown	Indeterminate	^e^
Immune thrombocytopenic purpura	1	M	4 years	Upper respiratory tract infection	Yes	19 days	Yes	Not recovered	Indeterminate	Haematologic system disorder and von Willebrand disease excluded
**Neurological diseases** ^a^										
Guillain–Barré syndrome	1	F	2 years	NA	No	< 1 day	No	Recovered	Inconsistent	No comments
	2	M	37 years	NA	NA	NA	No	Recovered	Inconsistent	History of mycoplasmal pneumonia
Paralysis	1	M	40–45 years	NA	NA	< 2 months	NAP	Unknown	Unclassifiable	No comments
Epilepsy	1	M	9 years	Preterm birth, symptomatic epilepsy, general developmental delay	No	< 1 day	NAP	Recovered	Indeterminate	Lowest Level Term coded as epilepsy aggravated

BC: Brighton Collaboration; F: female; M: male; NA: not available; NAP: not applicable.

^a^ According to a list of autoimmune and neurological diseases published by Arnheim-Dahlström et al. in 2013 [11]. Data not shown for autoimmune and neurological diseases on this list were without notifications for 4CMenB.

^b^ Causality of an AEFI was assessed according to the revised World Health Organization (WHO) classification [13].

^c^ Same patient.

^d^ Same patient.

^e^ Same patient.

### Serious events of specific interest

We also looked at some rare serious events of specific interest: febrile convulsion/seizure, anaphylactic reaction/anaphylactic shock, hypotonic-hyporesponsive episode and apparent life-threatening event. The only PRR that was significantly increased was found for febrile convulsion (n = 12; PRR 5.51 (95% CI: 3.06–9.92); chi-squared = 40.74).

#### Febrile convulsion/seizure

There were 12 case reports, eight infants and toddlers less than 2 years of age and four children 3 to 5 years of age, notifying ‘febrile convulsion’, 10 of which were assessed as ‘consistent’ and two as ‘inconsistent’ with a causal association to immunisation.

In addition, eight ICSR, three infants and toddlers less than 2 years of age, four children between 2 and 6 years of age and one adult, were received notifying ‘seizure’. Four ISCR were assessed as ‘inconsistent’ and one as ‘consistent’ with a causal association to immunisation. Causality of two ISCR was rated as ‘indeterminate’ and another one was assessed as ‘unclassifiable’.

Three of the 20 ICSR notifying febrile convulsion/seizure met the Brighton Collaboration case definition for convulsive seizure as an AEFI [14].

#### Anaphylactic reaction/anaphylactic shock

There was one ICSR with the coded PT ‘anaphylactic reaction’ and ‘anaphylactic shock’ referring to a female child 11 years of age without known pre-existing allergies. This case did not fulfil the criteria of the Brighton Collaboration case definition [15]. Causality was assessed as ‘unclassifiable’.

#### Hypotonic-hyporesponsive episode

There were three notifications of a hypotonic-hyporesponsive episode: one in a female infant 10 weeks of age, a female infant 6 months of age and a female toddler 13 months of age. All ICSR notifying hypotonic-hyporesponsive episode fulfilled the criteria of the Brighton Collaboration case definition [16] and all were rated as ‘consistent’ with a causal association to immunisation.

#### Apparent life-threatening event

There were two ICSR notifying an apparent life-threatening event: one in a female infant 6 months of age and one in a female infant 9 months of age. Both events resolved. In both ISCR notifying apparent life-threatening event, causality was rated as ‘unclassifiable’.

## Discussion

Results from this investigation largely correspond to reactogenicity findings from phase 2/3 clinical trials of 4CMenB including a safety population of 8,776 subjects from 2 months of age who received at least one dose of 4CMenB [17-23]. In general, the AEFI reports are consistent with the known safety profile of 4CMenB as reflected in the Summary of Product Characteristics which is dominated by administration site reactions. Analyses stratified by SOC and age group as well as SOC and sex did not reveal any age- or sex-related safety signals. Nevertheless, our analyses revealed some age-related differences within SOCs, e.g. a higher percentage of body temperature conditions/seizures in infants and toddlers compared with older individuals, which can easily be explained by age-specific background incidence rates. Regarding headaches, which were more frequently reported in adolescents and adults compared with younger individuals, this may be ascribed to the fact that infants and smaller children may be unable to report and/or correctly localise pain.

Screening of patient records for immune-mediated and neurological diseases according to an outcome list published by Arnheim-Dahlström et al. [11] did not generate any safety signal in terms of increased PRR. Continuous monitoring is considered sufficient for unexpected AEFI not listed in the Summary of Product Characteristics such as Henoch-Schoenlein purpura and myositis. In a published case of myositis after receipt of 4CMenB [24], magnetic resonance imaging suggested an incorrect placement of the vaccine into the shoulder joint or the shoulder bursa. This may have contributed to the development of a condition called ‘shoulder injury related to vaccine administration’ [25]. Injecting vaccine into the synovial tissue of the joint or bursa may cause severe inflammation. Notably, this case reveals off-label use as according to the Summary of Product Characteristics, in infants, the vaccine should be administered into the vastus lateralis muscle. In the other paediatric case report, myositis may have developed as a result of a hygiene problem since a smear test was positive for *Staphylococcus aureus*. The adult patient case report also involved off-label use and lacked information on how diagnosis of myositis was confirmed.

There were a total of 20 ISCR notifying seizures with and without fever (listed in the Summary of Product Characteristics as uncommon, i.e. ≥ 1/1,000 to < 1/100 adverse reaction). Only three of the 20 case reports met the Brighton Collaboration case definition for convulsive seizure as an AEFI [14]. This may be because the Brighton Collaboration case definition requires ‘a witnessed loss of consciousness’ or a ‘history of unconsciousness’ and that in most of the ISCR, ‘unconsciousness’ was not included in the description of signs and symptoms. Of note, a significantly increased PRR was found for ‘febrile convulsion’ but not for ‘seizure’ without documented fever. This is in line with the significantly increased PRR for pyrexia which is listed in the Summary of Product Characteristics as a very common (≥ 1/10) adverse reaction.

In point 4.5 of the Summary of Product Characteristics of 4CMenB, prophylactic use of paracetamol is recommended to reduce the incidence and severity of fever because studies have revealed that paracetamol has this effect without affecting the immunogenicity of either 4CMenB or routine vaccines. While Public Health England recommended the prophylactic use of paracetamol for this vaccine [26], the German National Immunization Technical Advisory Group neither recommended nor discouraged such. A recent publication even reported an increase in accident and emergency presentations for AEFI after introduction of the 4CMenB in United Kingdom from September 2013 to August 2016 despite prophylactic use of paracetamol [27]. As the meningococcal B vaccination has not yet been introduced in the vaccination schedule and thus, the vaccination coverage is supposed to be low, there is currently no possibility to determine whether the situation is similar in Germany. The high reactogenicity of 4CMenB compared with other vaccines used for routine vaccination was also confirmed in a recent review on clinical experience with vaccines against group B meningococcal disease [28].

There were three notifications of hypotonic-hyporesponsive episode (listed in the Summary of Product Characteristics) and two of apparent life-threatening event (not listed in the Summary of Product Characteristics) for which the diagnosis of apparent life-threatening event was considered uncertain as both ICSR notifying apparent life-threatening event fulfilled the criteria of the Brighton Collaboration case definition for hypotonic-hyporesponsive episode [16]. Hypotonic-hyporesponsive episode is labelled for several childhood vaccines.

There was one notification of sudden unexpected death (SUD) referring to a male child 28 months of age with a family history of inherited arrhythmogenic disease (a distant relative with a heterozygous genotype). Molecular genetic analyses to confirm or exclude a congenital long QT syndrome in the patient were performed, but results were not provided to us. In-depth cardiological diagnostics of the child’s parents were without indication of channelopathies. A recent publication revealed that channelopathies are important causes of SUD in infancy [29]. Sanchez et al. [30] who investigated 789 consecutive cases of SUD in individuals below 50 years of age and included genetic analysis in the investigation, found cardiac disease to be the most important cause of SUD. Oshima et al. even suggested performing genetic screening in addition to biochemical and physiological screening during the neonatal period to identify individuals at risk of arrhythmia or metabolic disease; affected infants could thus be diagnosed and treated earlier, and many cases of SUD could be prevented [31]. When assessing the causality of AEFI, background incidence rates have to be considered. Winkel et al. [32] determined the incidence rate of SUD in individuals 1 to 18 years of age in Denmark to be 1.5 cases per 100,000 person-years, and the highest possible incidence rate of sudden cardiac death as 1.1 cases per 100,000 person-years. Risgaard et al. 2014 [33] determined the age-specific sudden cardiac death incidence rate of children 2 to 3 years of age in Denmark to be 0.5 cases per 100,000 person-years. Based on data for the year 2015 obtained from The Federal Statistical Office of Germany upon request, the causes of death coded as ICD-10 R96 (Other sudden death, cause unknown), R98 (Unattended death), R99 (Other ill-defined and unspecified causes of mortality), and I46 (Sudden cardiac death, so described) were reported for four, nine, 23 and three children aged 1 to 5 years, respectively. Considering that 2,868,825 children 1 to 5 years of age lived in Germany in 2015, this corresponds to an incidence rate for R96, R98, R99, and I46 of 0.1, 0.3, 0.8 and 0.1 cases per 100,000 person-years, respectively. In the light of the above, it is expected that coincident SUD case reports unrelated to vaccination will be notified.

### Strengths

A major strength of this work is that because of the reporting obligation of healthcare professionals and marketing authorisation holders, the database used in this analysis is the most comprehensive AEFI collection in Germany. In order to receive more detailed information, follow-up reports were requested in almost all serious ICSR on a routine basis. Case definitions established by the Brighton Collaboration, if available, were used to validate the diagnoses notified by the healthcare professionals regarding immune-mediated and neurological diseases, as well as adverse events of specific interest. This allowed for objectifying the information provided and enabling comparisons with previous and future investigations on the safety profile of 4CMenB. In addition, we strictly adhered to the WHO criteria for causality assessment of AEFI which use Brighton Collaboration case definitions if applicable, overall scientific evidence and information concerning the individual case report.

### Limitations

Despite the legal obligation to notify AEFI, there is, of course, under-reporting and it is unclear to what extent. Meningococcal B vaccination has not yet been included in the national immunisation schedule and consequently, vaccine coverage data based on anonymised health insurance claims data were not available. The number of doses released was used as a surrogate for the number of doses administered. It has to be assumed that not all doses released were administered. Thus, the reporting rates presented within the scope of this work may be underestimated. Hence, for signal detection purposes we also used a disproportionality measure which is not based on exposure.

## Conclusions

Vaccination against bacterial meningitis caused by *Neisseria meningitidis* serogroup B, in general, is well tolerated. We analysed data from post-marketing surveillance over a period of 3 years by strictly adhering to WHO criteria for causality assessment and combining these findings with results of reporting rates and disproportionality analyses. Post-marketing surveillance of vaccines in Germany did not indicate any emerging safety signal. Rather, results were consistent with the known safety profile of the 4CMenB.
